# Comprehensive Identification of Sexual Dimorphism-Associated Differentially Expressed Genes in Two-Way Factorial Designed RNA-Seq Data on Japanese Quail (*Coturnix coturnix* japonica)

**DOI:** 10.1371/journal.pone.0139324

**Published:** 2015-09-29

**Authors:** Kelsey Caetano-Anolles, Minseok Seo, Sandra Rodriguez-Zas, Jae-Don Oh, Jae Yong Han, Kichoon Lee, Tae Sub Park, Sangsu Shin, Zhang Jiao Jiao, Mrinmoy Ghosh, Dong Kee Jeong, Seoae Cho, Heebal Kim, Ki-Duk Song, Hak-Kyo Lee

**Affiliations:** 1 Department of Animal Sciences, University of Illinois, Urbana, IL 61801, United States of America; 2 Interdisciplinary Program in Bioinformatics, Seoul National University, Kwan-ak St. 599, Kwan-ak Gu, Seoul, South Korea 151–741, Republic of Korea; 3 Department of Animal Biotechnology, Chonbuk National University, Jeonju 561–756, Republic of Korea; 4 Department of Agricultural Biotechnology, Animal Biotechnology Major, and Research Institute for Agriculture and Life Sciences, Seoul National University, Seoul 151–921, Republic of Korea; 5 Department of Animal Sciences, The Ohio State University, Columbus, OH 43210, United States of America; 6 Faculty of Animal Science and Biotechnology, Kyungpook National University, Sangju 37224, Korea; 7 Department of Animal Biotechnology, Faculty of Biotechnology, Jeju National University, Jeju 690–756, Republic of Korea; 8 CHO&KIM genomics, Main Bldg. #514, SNU Research Park, Seoul National University Mt.4-2, NakSeoungDae, Gwanakgu, Seoul 151–919, Republic of Korea; Chinese Academy of Fishery Sciences, CHINA

## Abstract

Japanese quail (*Coturnix coturnix* japonica) reach sexual maturity earlier, breed rapidly and successfully, and cost less and require less space than other birds raised commercially. Given the value of this species for food production and experimental use, more studies are necessary to determine chromosomal regions and genes associated with gender and breed-differentiation. This study employed *Trinity* and *edgeR* for transcriptome analysis of next-generation RNA-seq data, which included 4 tissues obtained from 3 different breeding lines of Japanese quail (random bred control, heavy weight, low weight). Differentially expressed genes shared between female and male tissue contrast groups were analyzed to identify genes related to sexual dimorphism as well as potential novel candidate genes for molecular sexing. Several of the genes identified in the present study as significant sex-related genes have been previously found in avian gene expression analyses (*NIPBL*, *UBAP2*), and other genes found differentially expressed in this study and not previously associated with sex-related differences may be considered potential candidates for molecular sexing (*TERA*, *MYP0*, *PPR17*, *CASQ2*). Additionally, other genes likely associated with neuronal and brain development (*CHKA*, *NYAP*), as well as body development and size differentiation (*ANKRD26*, *GRP87*) in quail were identified. Expression of homeobox protein regulating genes (*HXC4*, *ISL1*) shared between our two sex-related contrast groups (Female Brain vs. Male Brain and Ovary vs. Testis) indicates that these genes may regulate sex-specific anatomical development. Results reveal genetic features of the quail breed and could allow for more effective molecular sexing as well as selective breeding for traits important in commercial production.

## Introduction

Japanese quail (*Coturnix coturnix* japonica) have the fastest growth rate of all species in the Phasianidae family of birds, which includes species bred and raised primarily for human consumption such as chickens and turkeys [1]. Females reach sexual maturity at around 5–6 weeks on average, which is extremely early compared to the five-month maturation period of chickens. These features make the breed attractive not only for commercial production and genetic improvement, but also for research on reproduction and sex [2]. The feed and space required per bird is less for quail than for chickens or turkeys, making them excellent for commercial purposes [3]. In addition, the breed is valuable for experimental purposes [4]. Quail are commonly used as an experimental model for other species of commercial and non-commercial poultry and share many characteristics and behaviors with domestic chicken [5]. The birds lay eggs almost indefinitely under photoperiods longer than ~12 h and possess the ability to adapt and breed successfully in laboratory conditions [6]. Domestic quail produce eggs equivalent to their body weight every 19 days, with an egg-to-body weight ratio higher than that of the chicken. Females are reported to reach sexual maturity at 40 days, have a growing mortality of 5–20 percent, hatch ability of 60–70 percent, a fertility of 75–90 percent, and have a productive life of one year.

Given the demand for quail for commercial production and experimental use, more studies are necessary to determine chromosomal regions and genes associated with gender and breed-differentiation in the species. There are many biological strategies for understanding and using genetic molecular information. Two major approaches to comprehensive transcriptome profiling exist—microarray and next generation sequencing (NGS)-based RNA-seq. The microarray chip-based approach is the widest used for identifying numerous genes, simultaneously. Several exploratory gene expression studies have been performed on quails using microarrays [7,8]. However, this method does not provide a quantitative assessment of transcript abundance, which could affect the appropriate identification of suitable gene candidates for sexing. In turn, RNA-seq methods have recently become more popular for this line of research, and several comparative studies of the two approaches have shown that RNA-seq approaches are both more reliable and reproducible [9]. While many studies are routinely performed using RNA-seq, there have been no previous attempts to profile the quail transcriptome. This may be due to the absence of an official quail genome with transcriptome annotation that can be used as reference for transcript NGS sequence assembly, which involves mapping the overlapping reads that are generated onto reference genomic sequences. While official reference genomes and transcriptomic information exist on public databases for avian species such as zebra finch and chicken, only a recently developed DNA reference genome exists for quail [10]. In cases like this, a Trinity read assembly-based approach is suggested for the analysis of RNA-seq data without reference genomic and transcriptomic information [11].

In this paper, comprehensive RNA-seq profiling of Japanese quail was performed using 12 samples that include two factors (different quail lines and tissues) and a two-way factorial analysis. Model-based methodology has been developed for the RNA-seq which makes it possible to consider several related experimental factors [12]. By considering multiple factors, new undiscovered genes can be detected. Here, Trinity and *edgeR* were used to analyze transcriptome data obtained from 4 tissues [male brain (MB), female brain (FB), ovary (O), and testis (T)] and 3 different lines of Japanese quail (random bred control [RBC], low weight [LW], and heavy weight [HW]) with the initial goal of identifying significant sex related genes.

## Material and Methods

### Animal handling and tissue sampling

Samples were collected from 12 total animals, 4 from each of the three Japanese quail breeding lines: randomly bred control (RBC), heavy weight (HW), and low weight (LW). These lines were selectively bred over 80 generations for their body weight. Individuals were reared at The Ohio State University Poultry Facility, Columbus, OH. The animal care and experimental procedures followed protocol (2013A00000041) approved by the OSU Institutional Animal Care and Use Committee. Individuals were housed in cages specifically designed for quail with light provided 14 hours a day. Quail were sacrificed at 3-months of age during their light cycle. Samples from the brain, testis, and ovary were collected, snap-frozen in liquid nitrogen, and stored in a freezer at -80 degrees for processing. To extract total RNA from the ovary, large yolks were removed before homogenization. The whole testis and brain were used to extract total RNA.

### Procedures of RNA sample preparation

To generate RNA-seq reads, we applied Illumina-based NGS sequencing. Total RNA was prepared from tissues, quantitated using Nanodrop spectrophotometer (Thermo Scientific), and quality-assessed with the RNA 6000 Nano assay kit (Agilent) using a Bioanalyser 2100 (Agilent). NGS sequencing libraries were generated from one microgram of total RNA using the Truseq RNA Sample Prep Kit (Illumina) according to the manufacturer's protocol. Briefly, RNA was purified using oligo-T attached magnetic beads. After purification, the total poly A+ RNA was fragmented into small pieces (the average insert size for paried-end libraries was approximately 350bp). The segmented mRNA fragments were reverse transcribed. These fragments were purified with a QiaQuick PCR extraction kit, resolved with EB buffer, and linked to sequencing adapters. Adjoining distinct MID tags separated each cDNA library. The resulting libraries were then paired-end sequenced (2x101bp) with the Illumina HiSeq-2000 system. Finally, complete paired-end sequences were obtained as individual fastq files (forward and reverse) from output images with the CASAVA V 1.8.2 base calling software (https://support.illumina.com/sequencing/sequencing_software/casava.html) with ASCII Q-score offset 33.

### Read assembly pipeline

Unfortunately, no official reference genome for Japanese quail exists. In this situation, there are effectively two representative approaches of estimating gene expression using RNA-seq. One approach is to map reads to a related species. In the case of quail, the closest reference genome would be that of the chicken. This approach can only measure conserved regions in both species and is therefore not suitable for precisely profiling the quail’s transcriptome. The second, and more suitable, approach when a reference genome is not available is to reconstruct the genome/transcriptome by assembling reads to one another, creating a *de novo* assembly. We employed the Trinity pipeline for ‘non-model’ organisms, mapping the reads into assembled consensus without genome reference for measurement of transcriptome levels. Trinity enables the identification of transcript isoforms in non-model species. Prior to assembly, we employed Trimmomatic [13] for removal of adapter sequences. Next, we performed assembly and mapping with the following specific parameters: (1) In order to make reference consensus and assemble the transcriptome, we combined left and right reads, respectively, in paired-end reads using 12 samples; (2) Using combined left and right reads, we created the assembled transcriptome by Trinity.pl with ‘min_ker_cov_2’ option given the large number of reads; (3) The read mapping onto the assembled transcriptome was performed using bowtie2 [14] through the tophat2 interface [15]; (4) For the abundance estimation, we employed the RSEM tool [16]; (5) Gene annotation was performed using TransDecoder, implemented within Trinity [17]. Furthermore, we applied several developer-recommended gene annotation tools such as blastx, blastp, and hmmscan; (6) Using Trinotate, we extracted information of annotated genes with a default option. In order to define known transcriptomic regions, we used Trinotate (http://trinotate.github.io) with default options.

### Statistical analysis of RNA-seq data for detecting differentially expressed genes (DEGs)

In order to normalize gene expression in each sample, we used the TMM normalization method, implemented using edgeR [18]. We used a two-way factorial experimental design, in which 12 RNA-seq samples were obtained following two factors: tissue (FB, MB, O, and T) and line (RBC, LW, and HW). A two-way analysis of deviance (ANODEV) model has been shown to be effective in simultaneously considering the effect of factors for RNA-seq analysis. We applied this approach for our RNA-seq data. A more detailed statistical model is presented below:

$$\log{\left(\mu \right)}_{gj}=X_{i}^{T}\beta_{g}+\log N_{i},\;\text{with design matrix }X\text{ as follows:}$$ (1)

$$X=\left(\begin{array}{c} \begin{array}{c} \begin{array}{c} \begin{array}{cccccc} 1 & 0 & 0 & 0 & 0 & 0\\ 1 & 0 & 0 & 1 & 0 & 0 \end{array}\\ \begin{array}{cccccc} 1 & 0 & 0 & 0 & 1 & 0\\ 1 & 0 & 0 & 0 & 0 & 1 \end{array} \end{array}\\ \begin{array}{cccccc} 1 & 1 & 0 & 0 & 0 & 0\\ 1 & 1 & 0 & 1 & 0 & 0 \end{array} \end{array}\\ \begin{array}{cccccc} 1 & 1 & 0 & 0 & 1 & 0\\ 1 & 1 & 0 & 0 & 0 & 1 \end{array}\\ \begin{array}{cccccc} 1 & 0 & 1 & 0 & 0 & 0\\ 1 & 0 & 1 & 1 & 0 & 0 \end{array}\\ \begin{array}{cccccc} 1 & 0 & 1 & 0 & 1 & 0\\ 1 & 0 & 1 & 0 & 0 & 1 \end{array} \end{array}\right)$$ (2)

From left to right, columns represent intercept (baseline), LW, RBC, MB, O, and T, respectively. For easier understanding of the structure of explanatory variables, the linear predictor is simplified and represented as follows:

$$Expression_{ij}=\mu +Breed_{i}+Tissue_{j}$$ (3)

,where *i* = {"*RBC*", "*HW*", "*LW*"} and *j* = {"*FB*", "*MB*", "*O*", "*T*"}. As shown in (Eq 1) and (Eq 2), we applied a negative binomial-based GLM for detecting DEGs. In the design matrix (Eq 2), “HW” and “FB” are baseline. Therefore, by setting the values in the contrast matrix for interested parameters, we can perform statistical tests for detecting DEGs from certain hypotheses (i.e. using the contrast matrix = (1, -1, 0, 0, 0, 0), we can detect DEGs between HW vs. LW). We performed DEG tests with the following null hypotheses: (1) Effects of breeding line are zero; all organisms have identical gene expression; (2) Tissue effects are zero; all tissues have identical gene expression; (3) The difference between two specific groups is zero. These diverse statistical tests allowed comprehensive identification of DEGs in several conditions by considering two explanatory factors. The P-values from the whole tests on the model were adjusted by the Banjamini and Hochberg method [19] for controlling multiple test errors. In addition, we employed the DAVID functional annotation tool [20] for gene set enrichment analysis.

### Clustering analysis of RNA-seq data for investigating relationship of samples

In order to investigate the impact of tissue and line on gene expression and to understand the relationship between these two factors we employed hierarchical clustering, an unsupervised learning method, to unfold the structure of relationships among samples. In clustering analysis, the optimal *k* (number of clusters) is unknown. In the machine learning field, Silhouette scores have widely been used to estimate the optimal number of clusters [21, 22]. Generally, this score is determined by assuming that a well-organized cluster will have a smaller distance value within-cluster and a larger distance value in between clusters. We employed the *cluster* package within R to calculate the Silhouette score [23]; an individual score is generated for each sample, and we used an average of these scores to evaluate clustering. Many clustering methods for segregation of samples exist based on similarity such as K-means, hierarchical, etc.; for this study, we chose to use hierarchical clustering with two types of correlation based distance measures, Pearson and Spearman correlation coefficients, for understanding the similarity structure of the sample. While the Pearson correlation coefficient is widely used, it often results in different values depending on the distribution of the targeted two vectors. Therefore, we employed two alternate types of correlation measures in this study. In addition, when analyzing tree type similarity structure through hierarchical clustering, an agglomeration method should be used for calculation of the distance between clusters including several samples and the other cluster. We used an average method for determination of representative distance within clusters in this paper.

### Technical validation of DEGs detected from RNA-seq analysis using qRT-PCR

As RNA-seq data was generated under a non-biological replicated design, technical validation was performed in order to verify analysis results. We performed qRT-PCR experiments with 6 RBC-breed samples of brain tissue (n = 3, male and female, respectively) for 5 significantly detected DEGs- *CHD1*, *NIPBL*, *VIP*, *HXC4*, and *MYP0*. Total RNA was isolated from 4 different tissues using the TRIzol reagent (Invitrogen Carlsbad, CA, USA) according to manufacturer's instructions. We used Applied Biosystems Step-One Real-Time PCR system, EvaGreen dye (Biotium, Hayward, CA, USA) with β-actin (ACTB) as the control gene in order to determine relative gene expression (ΔCt). Primers for 5 DEGs are listed in S3 Appendix. In addition, a two-group t-test was employed to determine gene significance.

## Results

### De novo RNA-seq assembly and mapping of reads

A total of 534,610 transcriptome contigs were assembled using the Trinity pipeline. The average and median lengths of assembled contigs were 1,417 bp and 542 bp, respectively. There were 757,777,223 total assembled bases. The summary statistics of the reads aligned to assembled contigs is shown in Table 1. We generated assembled contigs with sufficient depth coverage using 12 whole samples without considering differences between breeding lines. This resulted in 4,502 annotated genes and associated transcription levels (read counts of known transcriptome regions) after filtering out non-expressed genes from all groups. In addition, we filtered results for transcripts having the highest length among duplicated annotated genes. A total of 3,503 unique annotated genes remained after filtering through this (above) criteria; these filtered genes were used in analyses to identify DEGs.

**Table 1:** Summary of RNA-seq samples and their mapping rate

Breed/Library	Reads #	Mapping rate by aligned reads on assembled contigs
RBC/FB	75,136,718	68.6%
RBC/MB	63,634,582	67.8%
RBC/O	35,523,482	68.6%
RBC/T	50,943,940	67.1%
LW/FB	48,319,732	74.6%
LW/MB	68,187,382	72.8%
LW/O	63,770,574	75.3%
LW/T	53,743,900	70.2%
HW/FB	40,652,156	67.7%
HW/MB	69,818,488	68.1%
HW/O	65,763,298	67.8%
HW/T	63,851,018	67.3%

### Significant genes between quail lines using contrast analysis upon two-way factorial designed model

**Fig 1:**
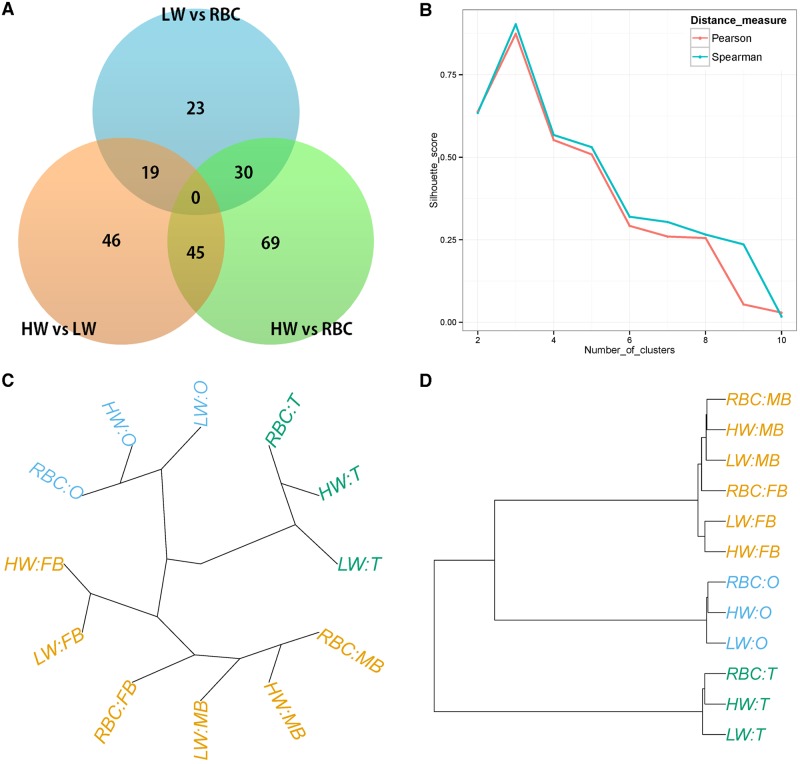
Comparison gene expression profiling results between the three breeds. (A) Venn diagram of DEGs used in pairwise statistical tests and contrast matrix of RBC, LW, and HW lines (Significance level: FDR-adjusted P-value <0.05). (B) Silhouette score plot for identification of optimal number of clusters. Red and blue colors represent distance measures; Pearson’s and Spearman’s correlation coefficients, respectively. (C, D) Based on the optimal number of clusters’ *k* = 3, gene expressions were visualized as radial and dendrogram types’ tree across hierarchical clustering analysis.

First, we identified DEGs among the RBC, LW, and HW breeding lines under the null hypothesis, $H_{0}:Breed=0$, for detection of DEGs differentially expressed in one or all lines. We identified a total of 205 significant (FDR-adjusted P-value < 0.05) DEGs. We then performed downstream analysis of assembled transcripts to explore the biological mechanisms of these DEGs. Results of gene-set clustering analysis using DAVID revealed significant enrichment of the extracellular matrix (ECM) related cluster, which includes extracellular region (3.1E-5), proteinaceous extracellular matrix (3.8E-3), and extracellular matrix (4.6E-3) pathways among others. To further investigate line differences, we performed pairwise comparison on the three quail lines using a contrast matrix with a GLM. We observed 102, 136, and 72 DEGs in the HW vs. LW, HW vs. RBC, and LW vs. RBC contrasts respectively (Fig 1A). Results of the pairwise test on the entire set of genes are listed in S1 Appendix. Here, we focused on the interpretation of difference between HW and LW lines, as these lines display a drastic phenotypic difference in terms of body size. Therefore, we expected detection of genes involved in regulating body size in the DE-list obtained from the comparison of HW and LW lines. We performed clustering analysis using *edgeR* on TMM-normalized values with whole genes in order to clarify the relationship between quail lines and tissue samples. The optimal number of clusters was estimated as 3 using the Silhouette scoring method with two types of correlation coefficients (Fig 1B). From this number, we visualized the tree with a cutoff of *k* = 3. HW and RBC lines have similar gene expression pattern compared to the LW line as shown in (Fig 1C and 1D). In addition, samples were clearly distinguished based on the tissue except for brain tissues; FB and MB. Across hierarchical clustering analyses, RBC-FB and RBC-MB were found to be clustered together. This indicates that gene expression is very similar in female and male quail brains. For more detailed examination of this phenomenon, we performed a statistical test for comparison between tissues.

### Detected DEGs tissues comparison

**Fig 2:**
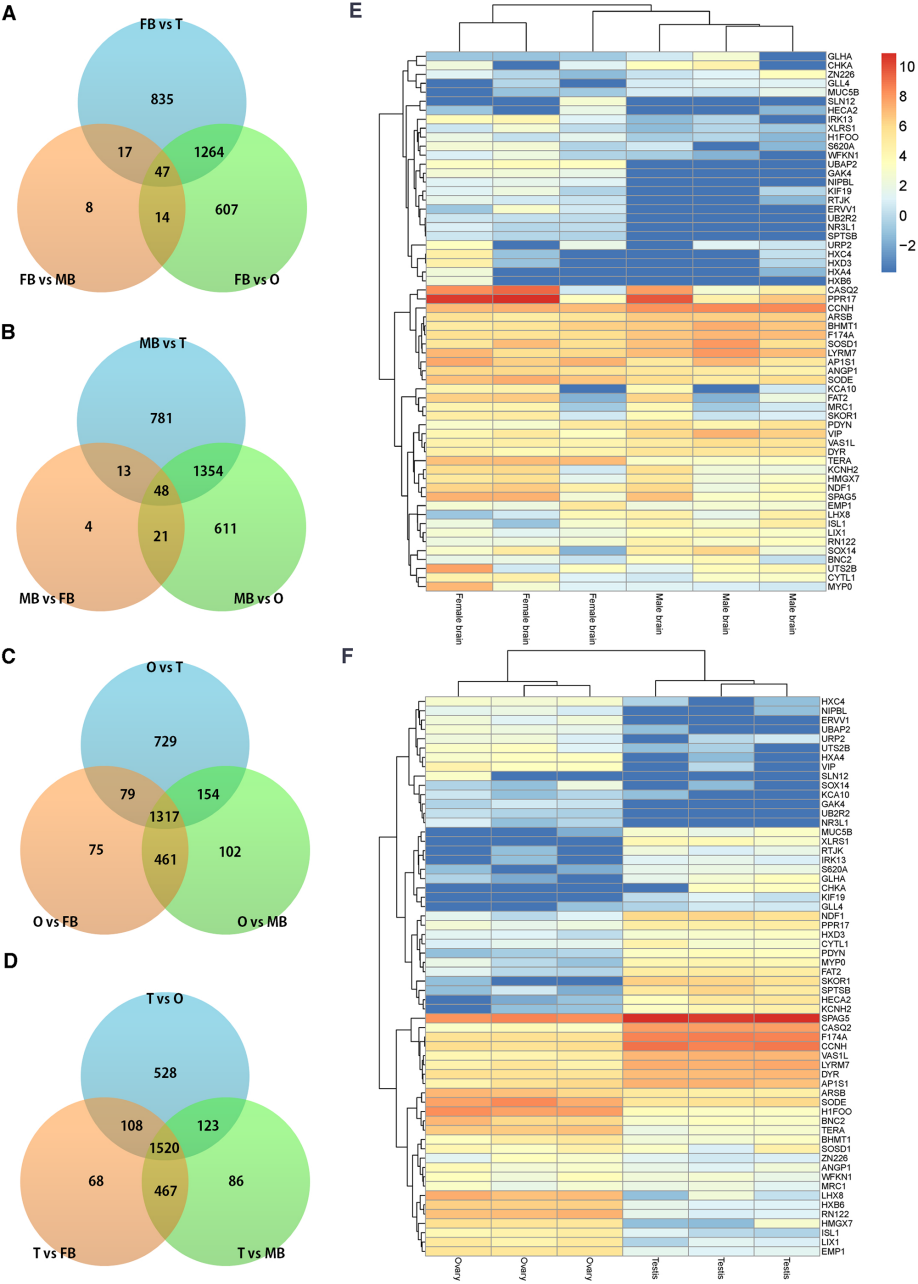
Comparison gene expression profiling results between different tissues. (A-D) Venn diagram comparing differentially expressed genes from pairwise statistical tests using a contrast matrix on four tissues (FB, MB, O, and T). Each value indicates mean number of significant genes in specified category. (E) Heatmap with 60 commonly identified DEGs in the FB vs MB and T vs O contrast, and 6 brain samples. (F) Heatmap with commonly identified 60 DEGs in FB vs MB and T vs O contrast, and 6 ovary and testis samples.

In order to investigate the tissues effect, we performed a GLM statistical test on the whole gene data. Results of this test revealed 86, 1932, 2163, 2034, 2196, and 2279 DEGs in the pairwise comparisons FB vs. MB, FB vs. O, FB vs. T, MB vs. O, MB vs. T, and O vs. T, respectively (S2 Appendix). Of the resulting DEGs, the most significant genes are shown in Table 2. We previously observed a less significant effect in the FB and MB contrast compared to the others through clustering analysis. For this reason, the number of DEGs identified between FB and MB is also smaller than that of other groups, as shown in (Fig 2A–2D). In addition, the comparison between O and T showed the greatest number of DEGs. In general, the effect of differences among tissues on gene expression was greater than that of differences between breeding lines. Although we ran all possible pairwise comparisons among all tissues, our focus is primarily on the sex-related difference between male vs. female brain (MB vs FB) and testis vs. ovary (T vs O). As shown in (Fig 2E and 2F), 60 genes were commonly identified as DEGs between the two contrasts. In this heatmap, we observed that very different gene expression patterns between MB vs MB or T vs O. These genes would be strong candidate genes for establishing an understanding of sex-differentiation in quail for purposes of molecular sexing, filling a gap in the research previously conducted on this species.

**Table 2:** Top 10 significant (FDR-adjusted P-Value < 0.05) DEGs shared between the Female Brain vs. Male Brain and Ovary vs. Testis contrast groups.

	FDR adjusted P-Value	
Gene symbol*	FB vs. MB	O vs. T
TERA	5.52E-23	1.35E-30
MYP0	3.43E-15	5.65E-14
UBAP2	3.20E-20	4.07E-11
VIP	1.35E-08	2.07E-22
PPR17	5.20E-08	2.64E-10
HXC4	1.53E-07	2.04E-13
ERVV1	7.76E-07	5.82E-12
CASQ2	1.80E-06	4.58E-28
NIPBL	3.30E-06	1.07E-07
ISL1	1.66E-05	8.35E-23

*Expanded gene names, listed in alphabetical order: CASQ2 = calsequestrin 2 (cardiac muscle); ERVV1 = Endogenous Retrovirus Group V, Member 1; HXC4 = Homeobox Protein CP19; ISL1 = ISL LIM Homeobox 1; MYP0 = myelin P0 protein; NIPBL = Nipped-B-like protein, also known as delangin; PPR17 = Pentatricopetptide repeat (PPR) protein 17; TERA = Valosin-containing protein; UBAP2 = ubiquitin Associated Protein 2; VIP = Vasoactive Intestinal Peptide.

### Technical validation of the detected sex-related DEGs using qRT-PCR

**Fig 3:**
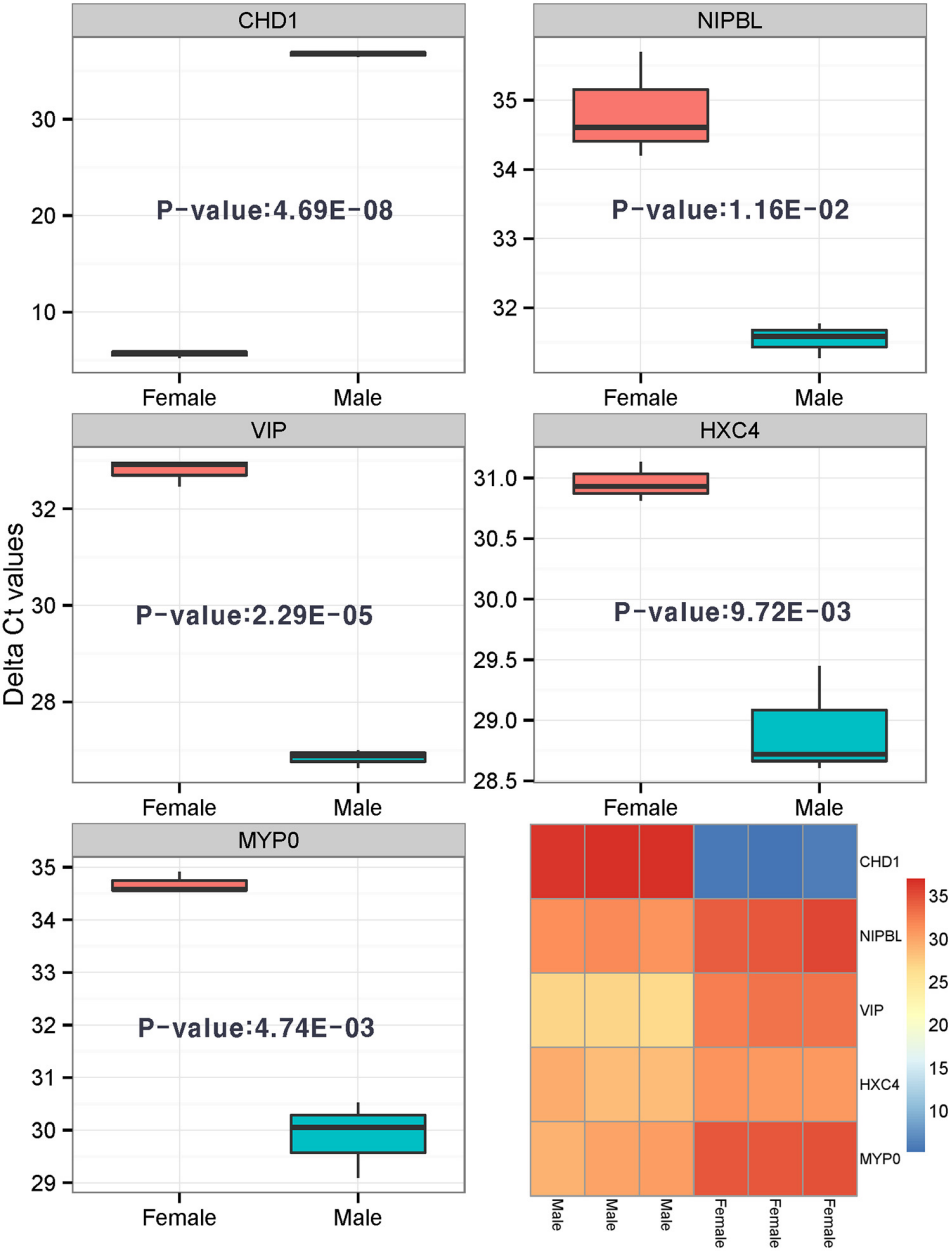
Verification of significantly detected sex-related DEGs using qRT-PCR. Five genes (*CHD1*, *NIPBL*, *VIP*, *HXC4*, *MYP0)* were successfully validated by qRT-PCR (P-value < 0.05). A two-group t-test was employed for statistical analysis of qRT-PCR data. Box-plots represent delta-ct values (control gene is beta-actin).

For verification of RNA-seq analysis results, we performed qRT-PCR on significantly detected genes between female brain vs male brain. A total of five genes were selected for analysis- *CHD* (FDR adjusted P-value: 0.008), *NIPBL* (3.30E-06), *VIP* (1.35E-08), *HXC4* (1.53E-07), and *MYP0* (3.43E-15) as shown in (S2 Appendix). All genes were found to also be significantly detected in qRT-PCR analysis (Fig 3). *CHD1* (P-value: 4.69E-08) was detected as over-expressed in MB. Another four genes; *NIPBL* (1.16E-02), *VIP* (2.29E-05), *HXC4* (9.72E-03), and *MYP0* (4.74E-03) were also significantly detected in a two group t-test (P-value < 0.05) based on the qRT-PCR results. Through these analyses, we confirmed that de novo based RNA-seq analysis was successfully performed on quail data.

## Discussion

### Sexual dimorphic genes in quail species

Analysis and interpretation of DEGs shared between female and male tissues (FB vs. MB and O vs. T) contrast groups provided insight into sex-related differences. Analysis therefore advanced the primary goal of this study—the identification of potential novel candidate genes for molecular sexing in quail. The link between *CHD1* and sex chromosomes has been well established. *CHD1* is used as a universal target for molecular sexing in birds, and has been studied extensively in multiple avian species [24, 25]. Two homologous *CHD1* genes, *CHD1*-W and *CHD1*-Z, have been mapped onto each W or Z chromosome, the heterogametic sex chromosomes of birds [26]. While sexing is typically performed through PCR-amplification of these two genes in many species of birds, several concerns regarding this method make identification of species-specific sex related genes particularly salient. Polymorphisms of *CHD* have been identified in auklets but in no other species; without knowledge of the existence of polymorphisms, the probability of incorrectly assigning sex is high [27]. *CHD1* did not appear as a significant differentially expressed sex-related gene in this analysis, further confirming that novel markers are needed for the quail breed. Several of the DEGs we identified as significant sex-related genes have been previously found in avian gene expression analyses.

UBAP2, a DEG shared between the Female Brain vs. Male Brain and Ovary vs. Testis contrast groups, has been found to be sex-related, specifically in chickens [28]. In a study of sexually dimorphic DEGs in chicken brains before gonadal differentiation, a UBAP2 gene located on chromosome W in the female embryonic brain was found up regulated. Other presumably sex-related genes have been found in chickens by genetic mapping to sex chromosomes. The chicken genome assembly contains 8 genes on the W-chromosome with homologous sequences on the Z-chromosome- *ATP5A1*, *CHD1*, *HINT*, *KCMF1*, *NIPBL*, *SMADS*, *SPIN*, and *UBAP2* [29]. Recently, 2 other genes have been mapped to the W-chromosome in chickens as well—*ZFR* and *ZNF532* [30]. *NIPBL*, found to be sex-related in these studies on chickens, was also found as a differentially expressed gene with a statistical significance by the present analyses in quail. Apart from *UBAP2* and *NIPBL*, the other significantly expressed genes shared between our two sex-related contrasts have not yet been confirmed as linked to avian sexual dimorphism; we propose that these genes are specific to quail and can be used for species-specific sexing.

A recent study investigated the evolution of avian sex chromosomes using retroposon insertions and random insertion/deletions in order to reconstruct gametologous gene trees [31]. Trees were reconstructed from three sex-related genes to elucidate their evolution–*CHD1*, *NIPBL*, and ATP synthase a-subunit isoform 1 (*ATP5A1*). The goal was to determine when the Z and W chromosomes differentiated, as they are highly differentiated in most extant birds. *CHD1* was found to differentiate in the neognathae ancestors; meanwhile, divergence of the previously mentioned *NIPBL* occurred in the neoavian ancestor. In the third gene they analyzed, *ATP5A1* (not found significant in the present analyses), differentiation occurred independently in 3 different types of birds—chickens, the screamer, and duck-related bird ancestors. This research and analysis of divergence is important for knowing which genes can be used in sexing tests in particular species of birds. The three insertions postulated to cover almost all species of living birds. However, species-specific information for quail can allow for more accurate and accessible molecular sexing.

Two genes from the top list of significant DEG’s, *VIP* and *ERVV1*, appear to be linked to sex and sexual dimorphism in mammals, although no previous research has been performed in the avian species. Gene *VIP* has been associated with sexual dimorphism in chickens [32] and rats [33, 34]. Although ERVV1’s function is unknown, placenta-specific expression has been observed, indicating that it may be sex-related [35]. Also interesting is the appearance of two homeobox protein-regulating genes in the top DEG list- *HXC4* and *ISL1*. Homeoboxes are DNA-sequences that regulate patterns of anatomical development; these results suggest that *HXC4* and *ISL1* may be responsible for anatomical sex differences between male and female quail.

### DEGs related to body size of quail lines, identified through comparison of HW and LW

Statistical comparison of the three breeding lines (RBC, LW, and HW) resulted in a total of 205 significant (FDR-adjusted P value < 0.05) DEGs. Gene ontology information on this gene list was obtained using DAVID. The ECM related cluster was significantly enriched in this analysis. In a previous transcriptomic study on chickens [36], comparison of microarray data from slow-growing and fast-growing birds revealed the significant impact of the ECM-receptor interaction pathway on chicken muscle development. The ECM-receptor interaction was expected to impact size differentiation in LW and HW quail, which may involve considerable genetic and regulatory changes at organismal level. Five genes were found to be significantly associated with the ECM-receptor interaction pathway: *CHAD*, *FINC*, *LAMC2*, *SV2C*, and *SDC4*. Knowledge of genes associated with size differentiation not only deepens our understanding of valuable genetic features of the quail species, which are not well understood, but also provides knowledge and potential for selective breeding strategies to increase body size and maximize profits in commercial production of quail.

Table 3 lists the top 10 most significant DEGs between HW and LW. The choline kinase alpha (*CHKA*) gene, only expressed in brain and testis, was significantly differentially expressed between HW and LW (Fig 2E and 2F). Previous studies have shown that this gene impacts neuronal differentiation, causing neurite outgrowth [37] as well as influencing development of the brain in rodent models [38]. Given these reports and our results, it appears that *CHKA* may play a significant role in brain size development in the quail species as well. Remarkably, the differentially expressed *ANR26* protein-coding gene (*ANKRD26*) has been highly associated with body size in mice [39, 40], making it a potential candidate gene for determining body development in quail and other bird species. The G protein-coupled receptor 87 (*GRP87*), a lysophosphatidic acid (LPA) receptor, was differentially expressed only in the testis tissue of LW quail. LPA affects skeletal development [41, 42]. *GPR87* may be also a potential candidate gene for regulating bone growth that eventually contributes to different body size between HW and LW, since these G coupled receptors prevent apoptosis and increase cell proliferation. *NYAP*, a gene that is typically expressed in developing neurons [43], was found differentially expressed between HW and LW in whole tissues. *NYAP* was found to be over-expressed in the brain of female LW quails compared to samples from both male and female HW quails. The number of differential expressed genes between HW and LW associated with brain and neuronal develop indicate that there may be several significant developmental differences between HW and LW varieties of quail which should be explored further.

**Table 3:** Top 10 most significant DEGs between HW and LW Quail.

Gene_Symbol*	logFC^	logCPM	Raw P-Value	FDR-Adjusted P-value
CHKA	-7.94168	2.47913	4.99E-21	1.75E-17
GPR87	5.724773	3.538677	6.78E-17	1.19E-13
NYAP1	6.82748	1.707929	1.00E-14	1.17E-11
MSLNL	5.720661	2.693912	3.78E-13	3.31E-10
ANR26	-3.82005	1.402079	1.15E-12	8.06E-10
OASL2	-2.2346	6.827207	1.51E-12	8.81E-10
IFI6	-2.15212	5.84676	9.03E-12	4.52E-09
T22D1	1.956184	5.098446	6.81E-11	2.72E-08
MTA70	-1.81969	6.818426	6.98E-11	2.72E-08
RBBP7	4.489682	1.741669	2.39E-10	8.37E-08

*The log2 fold change (log FC), log2 counts-per-million (logCPM), and P-values were calculated using edgeR.

^Expanded gene names, listed in alphabetical order: ANR26 = ANR26 protein-coding gene; CHKA = choline kinase alpha; GPR87 = G protein-coupled receptor 87; IFI6 = Interferon, Alpha-Inducible Protein 6; MSLNL = Mesothelin-Like; MTA70 = Methyltransferase Like 3; NYAP1 = Neuronal Tyrosine-Phosphorylated Phosphoinositide-3-Kinase Adaptor 1; OASL2 = 2'-5'-oligoadenylate synthase-like protein 2; RBBP7 = Retinoblastoma Binding Protein 7; T22D1 = Inosine-5'-monophosphate dehydrogenase

## Conclusion

In summary, two of the genes identified here as significant sex-related genes, NIPBL and UBAP2, have been established in previous studies as related to sex-differentiation in various avian species. The appearance of these genes as differentially expressed indicates that our contrast and experimental design allows for accurate identification of transcriptomic markers related to sex in quail. The other differentially expressed genes (e.g. *TERA*, *MYP0*, *PPR17*, *CASQ2*), which have not been previously linked to sex differentiation in neither avian nor other species, may be specific to sexual dimorphism in quail and should be further explored. Evolutionary analysis of the sex-related genes identified as significant can be used to study their history and determine when the Z and W chromosomes differentiated. Analysis of expression of homeobox protein regulating genes in the sex-related contrast group (Female Brain vs. Male Brain and Ovary vs. Testis contrast groups) reveals genes (e.g. *HXC4*, *ISL1*) which may regulate sex-specific anatomical development. In addition, and more importantly, genes can be used as tools for molecular sexing to improve streamlining of a process that is now used non-specifically for all birds but has potential for optimization and specification in quail.

### Accession Numbers

We generated raw RNA-seq and processed count data, which are available at GSE64961 in the GEO database.

## Supporting Information

S1 Appendix Output tissue. (TXT)

S2 Appendix Output breed. (TXT)

S3 Appendix Supplementary qRT-PCR. (PDF)

## References

[1] Ricklefs R. Patterns of growth in birds. II. Growth rate and mode of development. Ibis. 1973;115(2):177–201.

[2] Hinshaw W, Burmester B, Creamer A, Hess C, Howes J, Insko W, et al Coturnix (Coturnix coturnix japonica): standards and guidelines for the breeding, care and management of laboratory animals. National Academy of Sciences, Washington DC 1969.

[3] Wilson W, Anderson B, Siopes T. Importation of wild strain Japanese quail (wild coturnix) offers new game bird possibility. California Agriculture. 1971;25(7):5–6.

[4] Wilson WO, Abbott UK, Abplanalp H. Evaluation of Coturnix (Japanese quail) as pilot animal for poultry. Poultry Science. 1961;40(3):651–7.

[5] Mills A, Herron K, Bain M, Solomon S, Faure J, editors. Eggshell quality in Japanese quail Coturnix Japonica genetically selected for high or low levels of fearfulness Proceedings of the 9th European Poultry Conference, Glasgow, UK; 1994.

[6] TOUART L. REVISED DRAFT DETAILED REVIEW PAPER FOR AVIAN TWO-GENERATION TOXICITY TEST. 2003.

[7] Nadeau NJ, Minvielle F, Ito Si, Inoue-Murayama M, Gourichon D, Follett SA, et al Characterization of Japanese quail yellow as a genomic deletion upstream of the avian homolog of the mammalian ASIP (agouti) gene. Genetics. 2008;178(2):777–86. 10.1534/genetics.107.077073 18287407 PMC2248353

[8] Rawat A, Gust KA, Elasri MO, Perkins EJ. Quail Genomics: a knowledgebase for Northern bobwhite. BMC bioinformatics. 2010;11(Suppl 6):S13 10.1186/1471-2105-11-S6-S13 20946596 PMC3026360

[9] Marioni JC, Mason CE, Mane SM, Stephens M, Gilad Y. RNA-seq: an assessment of technical reproducibility and comparison with gene expression arrays. Genome research. 2008;18(9):1509–17. 10.1101/gr.079558.108 18550803 PMC2527709

[10] Kawahara-Miki R, Sano S, Nunome M, Shimmura T, Kuwayama T, Takahashi S, et al Next-generation sequencing reveals genomic features in the Japanese quail. Genomics. 2013;101(6):345–53. 10.1016/j.ygeno.2013.03.006 23557672

[11] Grabherr MG, Haas BJ, Yassour M, Levin JZ, Thompson DA, Amit I, et al Full-length transcriptome assembly from RNA-Seq data without a reference genome. Nature biotechnology. 2011;29(7):644–52. 10.1038/nbt.1883 21572440 PMC3571712

[12] Xiong Y, Chen X, Chen Z, Wang X, Shi S, Wang X, et al RNA sequencing shows no dosage compensation of the active X-chromosome. Nature genetics. 2010;42(12):1043–7. 10.1038/ng.711 21102464

[13] Bolger AM, Lohse M, Usadel B. Trimmomatic: a flexible trimmer for Illumina sequence data. Bioinformatics. 2014:btu170. 10.1093/bioinformatics/btu685 PMC4103590 24695404

[14] Langmead B, Salzberg SL. Fast gapped-read alignment with Bowtie 2. Nature methods. 2012;9(4):357–9. 10.1038/nmeth.1923 22388286 PMC3322381

[15] Kim D, Pertea G, Trapnell C, Pimentel H, Kelley R, Salzberg SL. TopHat2: accurate alignment of transcriptomes in the presence of insertions, deletions and gene fusions. Genome Biol. 2013;14(4):R36 10.1186/gb-2013-14-4-r36 23618408 PMC4053844

[16] Li B, Dewey CN. RSEM: accurate transcript quantification from RNA-Seq data with or without a reference genome. BMC bioinformatics. 2011;12(1):323. 21816040 10.1186/1471-2105-12-323 PMC3163565

[17] Haas BJ, Papanicolaou A, Yassour M, Grabherr M, Blood PD, Bowden J, et al De novo transcript sequence reconstruction from RNA-seq using the Trinity platform for reference generation and analysis. Nature protocols. 2013;8(8):1494–512. 10.1038/nprot.2013.084 23845962 PMC3875132

[18] Chen Y, Lun AT, Smyth GK. Differential Expression Analysis of Complex RNA-seq Experiments Using edgeR. 2014.

[19] Benjamini Y, Hochberg Y. Controlling the false discovery rate: a practical and powerful approach to multiple testing. Journal of the Royal Statistical Society Series B (Methodological). 1995:289–300.

[20] Da Wei Huang BTS, Lempicki RA. Systematic and integrative analysis of large gene lists using DAVID bioinformatics resources. Nature protocols. 2008;4(1):44–57. 10.1038/nprot.2008.211 19131956

[21] Gat-Viks I, Sharan R, Shamir R. Scoring clustering solutions by their biological relevance. Bioinformatics. 2003;19(18):2381–9. 14668221 10.1093/bioinformatics/btg330

[22] Lovmar L, Ahlford A, Jonsson M, Syvänen A-C. Silhouette scores for assessment of SNP genotype clusters. BMC genomics. 2005;6(1):35. 15760469 10.1186/1471-2164-6-35 PMC555759

[23] Maechler M, Rousseeuw P, Struyf A, Hubert M, Hornik K. Cluster: cluster analysis basics and extensions. R package version. 2012;1(2).

[24] Ellegren H. First gene on the avian W chromosome (CHD) provides a tag for universal sexing of non-ratite birds. Proceedings of the Royal Society of London Series B: Biological Sciences. 1996;263(1377):1635–41. 9025311 10.1098/rspb.1996.0239

[25] Griffiths R, Daan S, Dijkstra C. Sex identification in birds using two CHD genes. Proceedings of the Royal Society of London Series B: Biological Sciences. 1996;263(1374):1251–6. 8858876 10.1098/rspb.1996.0184

[26] Ellegren H. Hens, cocks and avian sex determination. EMBO reports. 2001;2(3):192–6. 11266359 10.1093/embo-reports/kve050 PMC1083846

[27] Dawson DA, Darby S, Hunter FM, Krupa AP, Jones IL, Burke T. A critique of avian CHD‐based molecular sexing protocols illustrated by a Z‐chromosome polymorphism detected in auklets. Molecular Ecology Notes. 2001;1(3):201–4.

[28] Lee S, Lee W, Shin J, Han B, Moon S, Cho S, et al Sexually dimorphic gene expression in the chick brain before gonadal differentiation. Poultry science. 2009;88(5):1003–15. 10.3382/ps.2008-00197 19359689

[29] Nam K, Ellegren H. The chicken (Gallus gallus) Z chromosome contains at least three nonlinear evolutionary strata. Genetics. 2008;180(2):1131–6. 10.1534/genetics.108.090324 18791248 PMC2567362

[30] Wahlberg P, Strömstedt L, Tordoir X, Foglio M, Heath S, Lechner D, et al A high-resolution linkage map for the Z chromosome in chicken reveals hot spots for recombination. Cytogenetic and genome research. 2007;117(1–4):22–9. 17675841 10.1159/000103161

[31] Suh A, Kriegs JO, Brosius J, Schmitz J. Retroposon insertions and the chronology of avian sex chromosome evolution. Molecular biology and evolution. 2011;28(11):2993–7. 10.1093/molbev/msr147 21633113

[32] Delfino KR, Southey B, Sweedler J, Rodriguez-Zas S. Genome-wide census and expression profiling of chicken neuropeptide and prohormone convertase genes. Neuropeptides. 2010;44(1):31–44. 10.1016/j.npep.2009.11.002 20006904 PMC2814002

[33] Gozes I, Werner H, Fawzi M, Abdelatty A, Shani Y, Fridkin M, et al Estrogen regulation of vasoactive intestinal peptide mRNA in rat hypothalamus. Journal of Molecular Neuroscience. 1989;1(3):55–61. 2642065 10.1007/BF02896857

[34] Lam KS, Srivastava G. Sex-related differences and thyroid hormone regulation of vasoactive intestinal peptide gene expression in the rat brain and pituitary. Brain research. 1990;526(1):135–7. 2078813 10.1016/0006-8993(90)90259-e

[35] Blaise S, De Parseval N, Heidmann T. Functional characterization of two newly identified Human Endogenous Retrovirus coding envelope genes. Retrovirology. 2005;2(1):19. 15766379 10.1186/1742-4690-2-19 PMC555746

[36] Cui H-X, Liu R-R, Zhao G-P, Zheng M-Q, Chen J-L, Wen J. Identification of differentially expressed genes and pathways for intramuscular fat deposition in pectoralis major tissues of fast-and slow-growing chickens. BMC genomics. 2012;13(1):213. 22646994 10.1186/1471-2164-13-213 PMC3420248

[37] Paoletti L, Elena C, Domizi P, Banchio C. Role of Phosphatidylcholine during Neuronal differentiation. IUBMB life. 2011;63(9):714–20. 10.1002/iub.521 21818839

[38] Craciunescu CN, Albright CD, Mar M-H, Song J, Zeisel SH. Choline availability during embryonic development alters progenitor cell mitosis in developing mouse hippocampus. The Journal of nutrition. 2003;133(11):3614–8. 14608083 10.1093/jn/133.11.3614 PMC1592525

[39] Bera TK, Liu X-F, Yamada M, Gavrilova O, Mezey E, Tessarollo L, et al A model for obesity and gigantism due to disruption of the Ankrd26 gene. Proceedings of the National Academy of Sciences. 2008;105(1):270–5. 10.1073/pnas.0710978105 PMC2224199 18162531

[40] Liu XF, Bera TK, Liu LJ, Pastan I. A primate-specific POTE-actin fusion protein plays a role in apoptosis. Apoptosis. 2009;14(10):1237–44. 10.1007/s10495-009-0392-0 19669888 PMC7285894

[41] Blackburn J, Mansell JP. The emerging role of lysophosphatidic acid (LPA) in skeletal biology. Bone. 2012;50(3):756–62. 10.1016/j.bone.2011.12.002 22193551

[42] Choi JW, Herr DR, Noguchi K, Yung YC, Lee C-W, Mutoh T, et al LPA receptors: subtypes and biological actions. Annual review of pharmacology and toxicology. 2010;50:157–86. 10.1146/annurev.pharmtox.010909.105753 20055701

[43] Yokoyama K, Tezuka T, Kotani M, Nakazawa T, Hoshina N, Shimoda Y, et al NYAP: a phosphoprotein family that links PI3K to WAVE1 signalling in neurons. The EMBO journal. 2011;30(23):4739–54. 10.1038/emboj.2011.348 21946561 PMC3243607

