# Supplementary material for: Comprehensive Identification of Sexual Dimorphism-Associated Differentially Expressed Genes in Two-Way Factorial Designed RNA-Seq Data on Japanese Quail (Coturnix coturnix japonica)
Source: PLoS One. 2015 Sep 29;10(9):e0139324. doi: 10.1371/journal.pone.0139324 (PMC4587967; doi:10.1371/journal.pone.0139324)
Supplement: S3 Appendix — (PDF) [file pone.0139324.s003.pdf]

Table 1. Used primers for qRT-PCR experiments

Gene symbol	Primers	Strand	Product size
**CHD1**	CTGAAAGGGAACAGCTGGAA	+	165
	GCAACTTCCTGGCATCAAAT	-	165
**NIPBL**	CAGAAGCTCTGGTTCACTCC	+	197
	TCAACAAGCTGAGTACAGGC	-	197
**VIP**	GATGGCTGTAGACACTGGTG	+	166
	GATGCAGCCAGTGAATCTGA	-	166
**HXC4**	ATGGGTTGTTTGGATGCTGT	+	161
	CAAACTACAGGAGGCCAACA	-	161
**MYP0**	CAGGAACGGAGCAGGTAAAA	+	159
	CAAGAACCCTCCTGACATCG	-	159

Table 2. Primer details of Beta actin

Primer	Sequence 5'- 3'	Size	Tm	NCBI
Beta actin LP	CGGTCAAGTCATCACCATTG	197	54 C	AF199488.1
Beta actin RP	CACTGCCAGGGTACATTGTG			

Table 3. The CT values of the qRT-PCR in the brain tissues of RBC breed

Target Name	Sample Name	CT	CT Mean
CHD1	Male	36.4434967	36.75757
	Male	36.8247514	
	Male	37.00446701	
	Female	5.796898365	5.640858
	Female	5.245229721	
	Female	5.880445957	
NIPBL	Male	31.58646393	31.54436
	Male	31.76962852	
	Male	31.27700043	
	Female	34.20628357	34.83852
	Female	34.60860825	
	Female	35.70066071	
VIP	Male	27.00	26.84354
	Male	26.89391327	
	Male	26.63669586	
	Female	32.46709824	32.78859
	Female	32.92311859	
	Female	32.97555161	
HXC4	Male	29.44915581	28.92547
	Male	28.71982193	
	Male	28.60742378	
	Female	31.13496017	30.96037
	Female	30.81520653	
	Female	30.93094444	
MYP0	Male	29.0953331	29.88831
	Male	30.05055618	
	Male	30.51903343	
	Female	34.57344437	34.67983
	Female	34.54977798	
	Female	34.9162674	

M1 1 2 3 4 5 M2 6 7 8 9 10

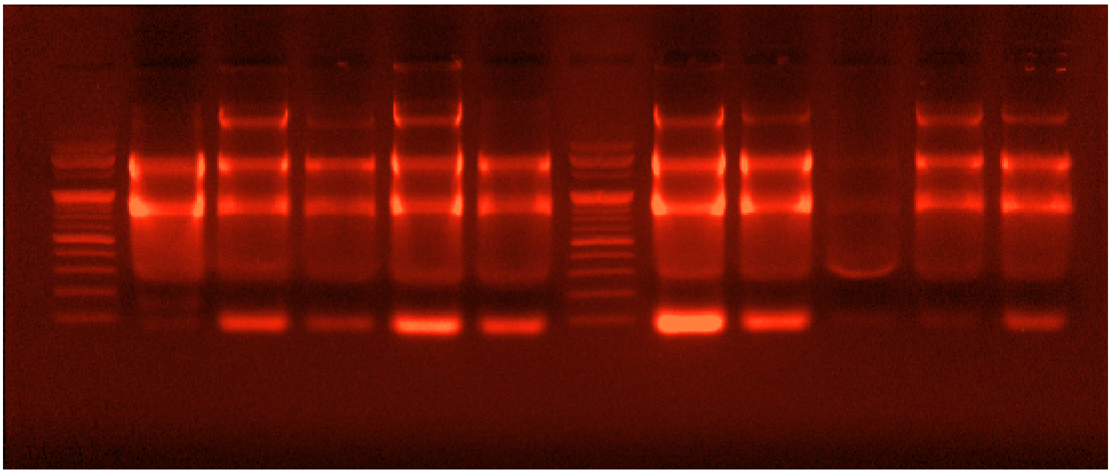

Fig.S1. RNA extraction from male and female brain samples.

M1 and M2: 100 bp marker
Load 1 to 5: Male samples
Load 6 to 10: Female samples

M1 1 2 3 4 5 6 7 8 9 10

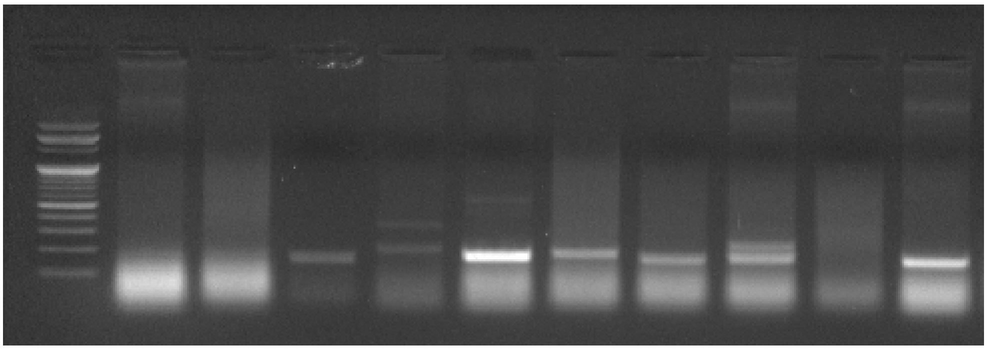

Fig.S2. Expression of genes in male and female brain samples (Using Eppendorf Master cycler gradient)

M1: 100 bp marker
Load 1 to 5: Male samples
Load 6 to 10: Female samples
[1 and 6: HXC4; 2 and 7: MYP0: 3 and 8: NIPBL; 4 and 10: VIP; 5 and 9: CHD1]
